# Use of 3D imaging for providing insights into high-order structure of mitotic chromosomes

**DOI:** 10.1007/s00412-018-0678-5

**Published:** 2018-09-03

**Authors:** Mohammed Yusuf, Kohei Kaneyoshi, Kiichi Fukui, Ian Robinson

**Affiliations:** 1 London Centre for Nanotechnology, University College London, London, WC1H 0AH UK; 2 Centre for Regenerative Medicine and Stem Cell Research, Aga Khan University, Karachi, 74800 Pakistan; 3 Graduate School of Engineering, Osaka University, Suita, Osaka 5650871 Japan; 4 Graduate School of Pharmaceutical Sciences, Osaka University, Suita, Osaka 5650871 Japan; 5 Brookhaven National Lab, Upton, NY 11973 USA

**Keywords:** Chromosome structure, Chromatin, 3D, High-resolution imaging

## Abstract

The high-order structure of metaphase chromosomes remains still under investigation, especially the 30-nm structure that is still controversial. Advanced 3D imaging has provided useful information for our understanding of this detailed structure. It is evident that new technologies together with improved sample preparations and image analyses should be adequately combined. This mini review highlights 3D imaging used for chromosome analysis so far with future imaging directions also highlighted.

## Chromosome structure

The DNA in chromosomes holds the genomic information of all eukaryotes. Throughout the cell cycle progression, chromosomes condense into the higher-order compact structures until they reach mitosis. The biological implications of this chromosome higher-order structure is for packaging the long DNA molecules into chromatin fibers within chromosomes facilitating upon cell division, the separation of the chromatids for transportation of DNA fibers evenly to two new daughter cells without damage (Sumner 2003). The higher-order structure of mitotic chromosomes has been under investigation for more than three centuries with the full structural details still remaining unknown. Our current understanding is that the 2-nm-thick DNA with 146 base pairs (bp) wraps twice around an octamer of histone proteins forming a series of nucleosomes. Chromatin is defined as complexes of nucleosomes and their associated proteins (Alberts et al. 2002). The X-ray crystal structure of the nucleosome has been resolved down to Angstrom resolutions (Luger et al. 1997; Davey et al. 2002) at 3.1 Å and tetranucleosome structure at 9 Å (Arents et al. 1991; Schalch et al. 2005). Recently, Ekundayo et al. published tetranucleosome structures at 5.8 and 6.7 Å (Ekundo et al. 2017). These nucleosomes form a “bead on a string” like structure having 11 nm diameter (Olins and Olins 1974). The binding of the linker histone (H1 or H5) organizes the nucleosome arrays into a more condensed 30-nm chromatin fiber (Robinson et al. 2006). Linker histone (e.g., H1 or H5) bound to a single nucleosome is known as a chromatosome and have shown to be involved in the 30-nm structure that is compacted further into a mitotic chromosome.

The 30-nm chromatin fibers have frequently been observed by scanning electron microscopy (SEM) (Inaga et al. 2008); several models for the folding of the nucleosomes have been proposed for the 30-nm fiber. However, many studies now even question the existence of this structure especially in vivo (Maeshima et al. 2010; Nishino et al. 2012a; Fussner et al. 2011). Many models have been proposed since the discovery of the one-start helix/solenoid model. This model was proposed after purifying the 10-nm fiber with a low concentration of cations that showed linear arrangement of the nucleosomes stacked with their neighbors (Finch and Klug 1976). The other model proposed is the zigzag or two-start cross-linker model where the nucleosomes are not stacked with their neighbors but zigzag back and forth to form a two-start stack of nucleosomes (Woodcock et al. 1984; Dorigo 2004). EM studies led onto a two-start flat ribbon model that had about 5 nucleosomes per 11-nm length (Dorigo 2004). A zigzag conformation was proposed after crystallizing tetranucleosomes and performing X-ray crystallography that led onto a model having a twisted ribbon with 24–25-nm approximated diameter and a compaction density of five to six nucleosomes per 11-nm (Schalch et al. 2005). Further EM studies including cryo-EM supported the one-start solenoid model that showed 30-nm measurements. The study showed that the linker histone H5 and the length of different nucleosome repeats had an effect on chromatin fiber architecture (Robinson et al. 2006). Recently, a cryo-EM study using 12 × 187 and 12 × 177 bp nucleosomes with reconstituted fibers in the presence of histone H1 has also shown repeating tetra-nucleosomal structural units supported the zigzag two-start helix model (Song et al. 2014). Scanning transmission electron microscopy (STEM) has been used on intact chromosomes showing chromatin fibers with a fibrous structure of 20 to 30-nm (Fukui 2016). With a large number of studies performed, the exact structure of the 30-nm fiber still remains unclear (Maeshima et al. 2016a). Most of these studies have been done in vitro and the existence of the 30-nm structure in vivo is debated. Several studies that do not support the 30-nm fiber existence (Maeshima et al. 2016a) include the small angle X-ray scattering (SAXS) studies done in solution that supports the 10-nm fiber (Joti et al. 2012; Nishino et al. 2012b). The disordered packing of 10-nm fibers has been suggested to be because of the face-to-face and edge-to-edge nucleosome–nucleosome interactions. This is known as the “polymer-melt” structure whereby the nucleosome fibers have been suggested to be moving and rearranging at the local level (Nishino et al. 2012b; Maeshima et al. 2014). Cryo-EM of frozen hydrated sections (close to native state) of chromosomes and nuclei has shown that the 30-nm exists in samples prepared after aldehyde fixation but not after cryo-preservation (Eltsov et al. 2014). Using electron spectroscopic imaging (ESI) with electron tomography showed 10-nm fibers in mouse euchromatic and heterochromatic regions (Fussner et al. 2012). Heterogeneous groups of nucleosomes called “nucleosome clutches” were visualized using super-resolution microscopy using mouse cells in situ and showed 10-nm fibers (Ricci et al. 2015). ChromEM that is a multitilt EM tomography and a labelling method that enhances the contrast of DNA showed that nucleosomes are organized into disordered chains. These have diameters ranging between 5 and 24-nm, highlighting that the 10-nm fiber is heterogeneous and varies in diameter (Ou et al. 2017). The 30-nm fiber structure formation is dependent on the condition the samples are prepared (Maeshima et al. 2016a). Divalent cations such as magnesium and calcium are involved in the chromatin organization and decondensation process (Dwiranti et al. 2016; Maeshima et al. 2016b; Phengchat et al. 2016). Compared to interphase nuclei, these cations are highly present in mitotic chromosomes. Lower concentrations of magnesium display the bead on a string like structure of 10-nm, but at higher concentrations, helical structures around ~ 30–40 nm in diameter have been observed (Dwiranti et al. 2016; Maeshima et al. 2018).

Proteins play an important role in the compaction of mitotic chromosome with over 158 proteins identified on chromosomes including the histones (Uchiyama et al. 2005; Ohta et al. 2010). The backbone structure of chromosomes after depletion of histone proteins is known as the chromosome scaffold (Paulson and Laemmli 1977). This consists of non-histone proteins, so-called scaffold proteins, which include condensin, topoisomerase IIα (Topo IIα), and kinesin family member 4 (Uchiyama et al. 2005; Earnshaw et al. 1985; Maeshima 2003; Samejima et al. 2012).

To fully understand the inner structure of the chromosome, both the chromatin fiber and the chromosome scaffold need to be understood (Fukui 2016). Different scientific approaches that have contributed hugely towards understanding how chromosomes are folded, compacted, and organized have been employed. Chromosome conformation capture techniques such as Hi C have been recently used to understand the contacts between chromatin fibers (Gibcus et al. 2018; Nagano et al. 2013; Naumova et al. 2013). Great efforts have also been made towards imaging chromosomes using different microscopy methods (Fukui 2016; Pollard and Earnshaw 2002; Flors and Earnshaw 2011). It is clear that three dimensional (3D) imaging is needed to study the intact mitotic chromosome. In this mini-review, we discuss the 3D imaging approaches used for investigating the higher-order chromosome structure. The limitations and future directions are also discussed.

## Chromosome sample preparation procedures

Mammalian or animal chromosomes are generally prepared from live cells after growing in cell culture (Howe et al. 2014). At metaphase, they are at the most compact state making them easier for isolation and analysis. Cells are treated with a mitotic inhibitor, e.g., colcemid or nocodazole (Moralli et al. 2011) that disrupts the spindle fibers. After treatment with a hypotonic solution such as KCl that swells the cellular volume, the sample is then fixed using methanol-acetic acid (MAA) that preserves the chromosomes (Tobias et al. 2011) but causes partial denaturation and precipitation of nucleic acid and proteins (Shihab 2012). Chromosomes can also be prepared using polyamine buffer containing spermine and spermidine that are stable in-solution and have been used for a number of applications (Yusuf et al. 2014a; Kannan and Zilfalil 2009; Takata et al. 2007).

## 3D imaging of chromosomes

Chromosome size, number, and morphology have been well characterized using standard 2D optical microscopy (Tobias et al. 2011). Banding methods allow investigation of structural alterations and numerical abnormalities (Kannan and Zilfalil 2009). G-banding that distinguishes the states of chromatin being heterochromatin and euchromatin can be identified showing as dark and light bands on the chromosome respectively (Tobias et al. 2011). The use of fluorescent dyes further allowed genes to be localized using fluorescence in situ hybridization (FISH), (Volpi and Bridger 2008). Determination of the higher-order mitotic chromosome structure using the optical microscope is not possible as the resolution is limited to the diffraction limit of light (~ 200-nm), making it difficult to resolve the smaller structures (Abbe 1873). This has made it difficult to study chromatin live cell structures at the nanoscale (Mora-Bermúdez and Ellenberg 2007). It has been reported that enhanced resolution can be beyond the Abbe diffraction limit of light (200-nm) with wavelength-scale solid immersion lenses (Mason et al. 2010) and through the assistance of InSb thin layers (Ding et al. 2016). Live cell studies use GFP to label the proteins of interest such as specific histones (Kanda et al. 1998) have been hindered over the years due to absence of methods for labelling of specific DNA sequences in morphologically intact chromosomes (Struckov and Belmont 2009). Recent studies have shown live cell imaging of a single labelled chromosome using CRISPR/Cas 9 technology (Zhou et al. 2017). This technology has also been used to multicolor label specific chromosomal loci in live cells (Ma et al. 2015).

Ultra-high-resolution microscopy has been used to investigate mitotic chromosomes at high resolution. Even though the transmission electron microscope (TEM) has Angstrom resolution, it is not a suitable microscopy for studying intact mitotic chromosomes because chromosomes are too thick for the electrons to penetrate through biological samples over 1 micron thick (Ris 1981). Whole human chromosomes have been imaged and reconstructed in 3D by TEM tomography after the sample was chemically prepared (Engelhardt 2000; Harauz et al. 1987). Forty-one projection images were obtained after tilting the sample from − 60° to + 60° to reconstruct. This led onto the reconstruction of a 3D chromosome in which internal structure showed 26 to 58-nm features and clearly showed distribution of 30-nm fiber that is consistent with the published looping model (Engelhardt 2000).

Scanning electron microscopy (SEM) images the sample surface by scanning it with a focused electron beam and collection of backscattered electrons. The major limitation has been due to sample preparation as MAA chromosomes show an artificial surface “skin” layer over the chromosomes following air-drying (Shemilt et al. 2014). A “drop-cryo” method was developed for barley chromosomes that allowed routine investigation of the surface structure information. On the arms of the chromosomes, chromomeres of around 200–300-nm were observed that had a knot-like structure representing highly condensed regions while the centromeres showed parallel fibers (Wanner and Formanek 2000; Iwano et al. 1997). Removal of nucleoplasm from the surface of MAA human chromosomes has been investigated after treating the chromosomes using a commercial enzyme product, cytoclear (Shemilt et al. 2014).

**Fig. 1:**
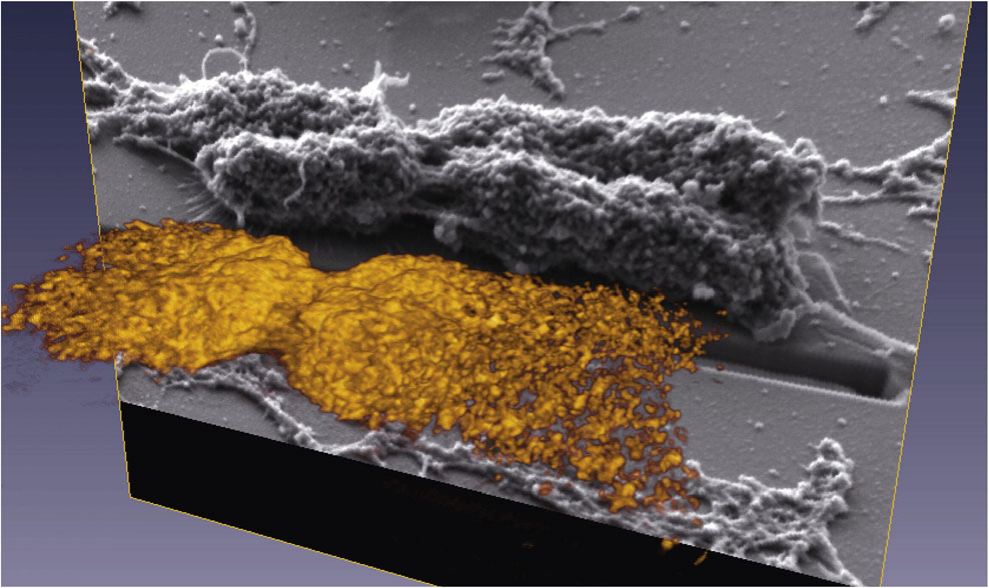
Reconstruction of a Barley chromosome after 3D FIB-SEM. 198 sections were aligned after imaging. The chromosome was immunogold labeled with phosphorylated histone H3 (ser 10) (yellow) (Schroeder-Reiter et al. 2009).

Barley chromosome structure has been investigated after focused ion beam SEM (FIB-SEM) (Schroeder-Reiter et al. 2009). After performing the drop-cryo method, the samples were critical-point dried. Platinum blue staining was performed, and the phosphorylated histone H3 (serine 10) was labelled with immunogold. Slices were taken using a Ga^+^ beam from the sample surface after it was tilted 54°. The dissected chromosome showed many cavities within the cross section. A full 3D-reconstructed chromosome was achieved from a series of 198 cross sections at resolutions between 1.5 and 3-nm but blurred by a labelling diameter between 15 and 30-nm (see Fig. 1) (Schroeder-Reiter et al. 2009). This method has a great advantage to obtain images with high resolution brought from direct observation of exposed inner structure; however, sample damage caused by accelerated Ga^+^ beam cannot be ignored.

**Fig. 2:**
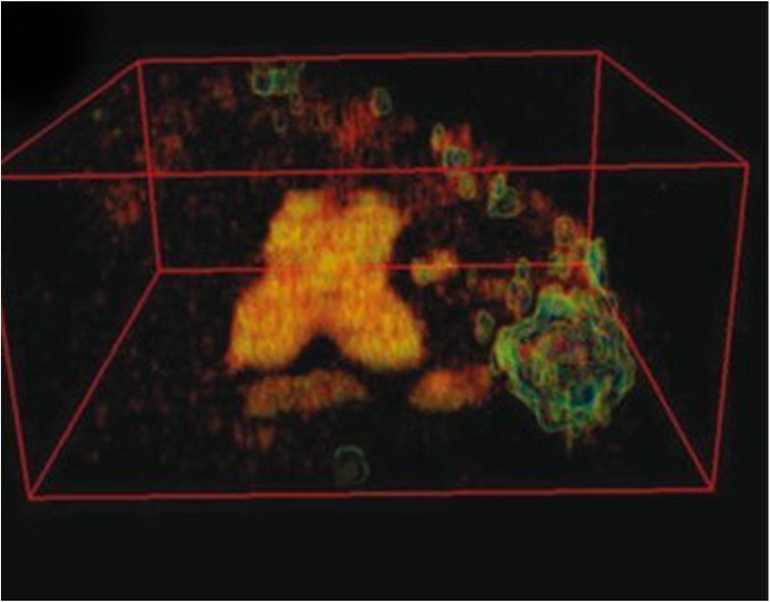
3D SBF-SEM of human mitotic chromosome. X-shaped human reconstructed after 13-nm × 100-nm sections. Box size 4.253 × 3.741 × 1.6 μm (extracted from (Yusuf et al. 2014b))

Serial block face SEM (SBF-SEM) allows 3D information and is similar to FIB-SEM but uses a diamond knife instead of focused G^+^ ions to cut the sample (Denk and Horstmann 2004). The sample is first embedded into a resin and dissected by ultra-microtome within the microscopy chamber, allowing serial inner structure images by SEM. Human chromosomes have been investigated using this method that allowed full 3D images of polyamine preparations (see Fig. 2) (Yusuf et al. 2014b). SBF-SEM has been used on a human prophase nucleus where 36 out of 46 were analyzed. Chromosomes showed porous network structure after MAA treatment and platinum blue staining. With a resolution of around 50-nm in 3D, the internal structure could not be determined; however, chromosomes showed parallel arrangement of chromatids. Sister chromatids showed curved cylindrical shapes with a well-conserved diameter of around 765 nm that were 2 to 3 μm long (Chen et al. 2017). A correlative approach known as 3D combined light microscopy and serial block face-scanning electron microscopy (CLEM) was used to study mitotic chromosomes. After analysis of wild-type and Ki-67-depleted chromosomes, it was shown that the periphery was 30–47% of the entire chromosome volume with more than 33% of the protein mass of isolated mitotic chromosomes determined by quantitative proteomics. This study concluded that the chromatin made a small percentage of the total mass of metaphase chromosomes (Booth et al. 2016). SBF-SEM and FIB-SEM both involve serial cutting of the sample that destroys the original sample. Both the diamond knife and Ga^+^ beam can leave an amorphous layer after sectioning and also leave damage.

**Fig. 3:**
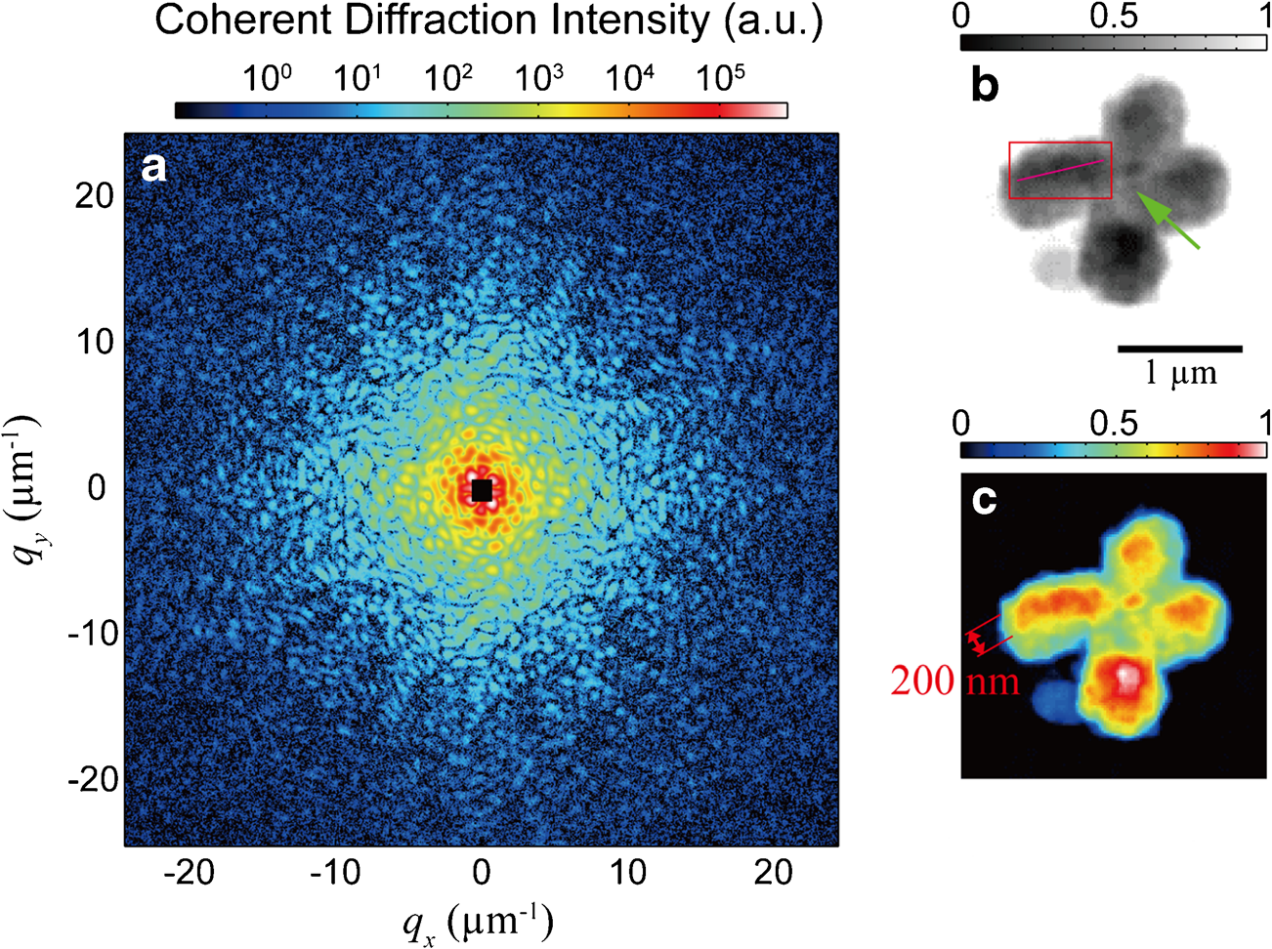
X-ray diffraction of unstained human chromosome with reconstructed projection image. **a** Coherent diffraction pattern of an human chromosome. Chromosome image **b** in gray scale with arrow indicating the centromere region. and Chromosome image **c** in color scale showing a high-intensity region on the chromatids  (extracted from (Nishino et al. 2009))

In contrast to TEM, X-rays have the ability to penetrate through whole intact chromosomes. Imaging intact chromosomes is advantageous and simplifies the procedure saving time for sample preparation. Coherent diffraction imaging (CDI) using X-ray diffraction was achieved on intact mitotic chromosomes in both 2D and 3D (Nishino et al. 2009). The sample preparation involved fixing intact chromosomes onto thin silicon membranes. Another huge advantage is that this study did not stain the sample with any heavy metals. The 2D resolution achieved was 38-nm. 3D was achieved after tilting the sample to different angles from − 70° to + 70° at intervals of 2.5° or 5°. For 3D data analysis, coherent diffraction data sets at 38 incident angles were used resulting in 120-nm spatial resolution in 3D. The study showed not only the surface of the chromosome but also high electron density around the centromere and chromosome axis. No significant internal structure was observed (see Fig. 3) (Nishino et al. 2009). This method has a disadvantage because the spatial resolution is often limited by the X-ray radiation damage and/or by statistical precision at high angles (Nishino et al. 2009).

Atomic force microscopy (AFM) may be another strong tool to visualize 3D chromosome structure. AFM gives us images which are similar to those obtained using SEM by scanning the solid sample surface with a sharp probe tip and detecting the interaction force between the tip and the sample to get height information. From this principle, AFM does not require metal coating and the observation can be performed either in vacuum, air, or liquid condition (Ushiki et al. 1996, 2008). Human chromosomes isolated by hexylene glycol method were dropped onto a glass slide and observed in hexylene glycol buffer. No fixation was performed. The obtained images showed 400–800-nm thick chromosomes whose surface was covered with globular or fibrous structures with about 50-nm in thickness (Hoshi et al. 2006).

**Fig. 4:**
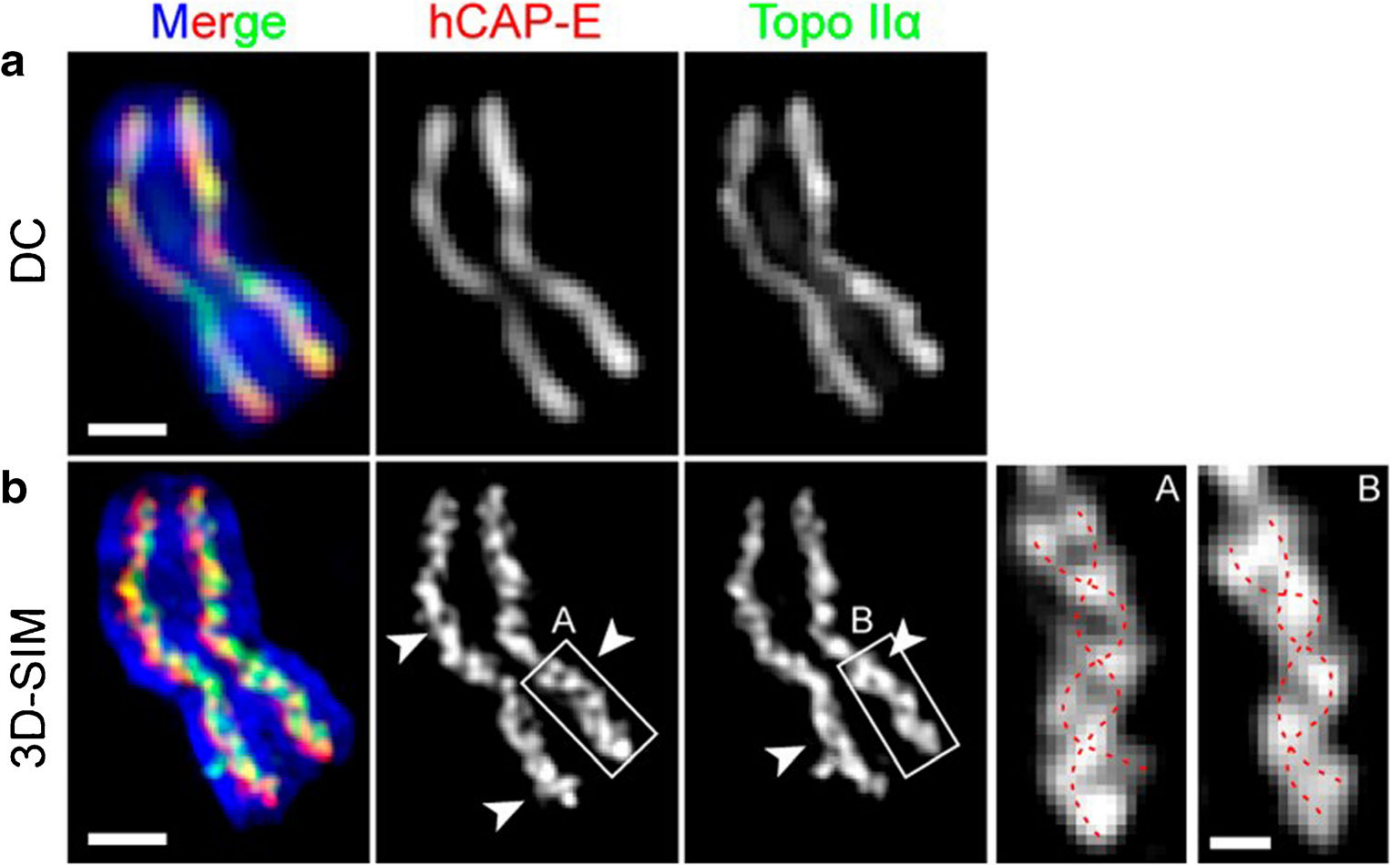
Imaging of metaphase chromosomes investigating double strands after immunostaining for hCAP-E and Topo IIα. **a** Wide-field microscopy after applying deconvolution (DC) of a immunostained chromosome shows difficulty in imaging the double strands. **b** 3D-SIM images of the same chromosome as a. Arrowheads show double stands. **a**, **b** Scale bars 1 μm. Insets A and B that are magnified views of **a** and **b** (white box) and clearly show double strands on the length of the chromosome that represents the chromosome scaffold (red dotted line). Scale bar for A and B insets is 250 nm (extracted from (Poonperm et al. 2015))

3D structured illumination microscopy (SIM), a super-resolution technique, has been performed on mitotic chromosomes (Carlton 2008). SIM allows us to get higher-resolution images by illuminating a sample with a striped pattern of visible light, which is rotated and scanned to give a number of images, obtained in different phases and direction conditions. After processing, the image resolution is said to reach approximately 120-nm (lateral), being twice the optical diffraction limit (Schermelleh et al. 2008). It has been shown that the axial distributions of scaffold proteins in metaphase chromatids are composed of two twisted double strands. It was suggested that this allows both chromosomal bending/flexibility and rigidity to occur (Fig. 4) (Poonperm et al. 2015).

## Future direction

Despite the impressive nature of imaging methods mentioned above, the detailed higher-order structure is still under debate. Optical super-resolution microscopy techniques are under development and present an opportunity to study the structure of the chromosomes below the diffraction limit. Such methods should allow the study of live cells that has not been possible with any previous high-resolution method and with development in 3D. Optical super-resolution techniques are already being applied to study chromatin contacts (Wang et al. 2017) and will prove useful for studying chromosomes. This technique is limited to the number of dyes used and the thickness of the sample. STEM allows visualization of 3D structures without sectioning the sample and does not require staining. The limitation is in the z-direction thickness (Fukui 2016).

3D studies done to date have tended to require harsh chemical fixation, dehydration, and drying steps that may not represent the true nature of the sample under investigation. Cryo-electron microscopy enables us to observe biological samples close to the native state (Eltsov et al. 2008). Chromosome samples are rapidly cooled down to cryogenic temperature that allows samples to be embedded in vitreous ice, which appears to be featureless. In principle, there is no morphology destruction caused by water volume expansion. Development of new technologies that will maintain the chromosome condition as close to its native state would be useful to obtain information of chromosome surface and interior details. Such methods would include 3D Cryo TEM, SEM, or X-ray imaging. To date, there is no report showing 3D metaphase chromosomes imaged with such techniques but these are under development. 3D Cryo FIB-SEM and Cryo 3D CDI have already been performed on cells (Schertel et al. 2013; Rodriguez et al. 2015), and Cryo X-ray Ptychographic imaging has been attempted on nuclei (Yusuf et al. 2017). Super resolution microscopy is proven promising for investigation chromatin structure (Sydor et al. 2015). Photoactivated localization microscopy (PALM) together with single molecule tracking has been applied to study chromatin dynamics in live cells that showed nucleosome domains during cell cycle (Nozaki et al. 2017). PALM has also been used to image *Drosophila* mitotic chromosomes at high resolution (∼ 30 nm) after labelling with H2AvD-EGFP, a histone H2A variant in the presence of background (Matsuda et al. 2010). Using new and advanced 3D imaging tools, the higher-order chromosome structure enigma should be resolved after a century of research.
